# A rare case of aggressive pyoderma gangrenosum with Cogan syndrome in a person with skin of colour

**DOI:** 10.1093/skinhd/vzae027

**Published:** 2025-02-14

**Authors:** Nageswary Nadarajah, Lucy Clark, Shernaz Walton

**Affiliations:** Department of Medicine, Hull University Teaching Hospitals NHS Foundation Trust, Hull, UK; University of Hull, Northern Lincolnshire and Goole NHS Trust, Scunthorpe, UK; Department of Dermatology, University of Hull, Hull University Teaching Hospitals NHS Foundation Trust, Hull, UK

## Abstract

Cogan syndrome (CS) is a rare autoimmune vasculitis affecting the audiovestibular and ocular systems. Its pathogenesis is unknown. CS was classified into typical and atypical CS in 1980 to aid its diagnosis. Its association with pyoderma gangrenosum (PG) has only been reported three times in the literature. This is also the first case of its occurrence in a person with skin of colour. CS is a diagnosis of exclusion and thus its diagnosis may present many challenges to healthcare professionals. Herein, we describe the case of a 75-year-old South Asian woman who presented acutely to the Stroke Unit following a right lacunar infarction which was treated with aspirin and clopidogrel. An enlarging nonhealing wound was noted at the site of a recent total left hip replacement. Intravenous antibiotics were started, with multiple surgical debridements performed. During admission, two new painful pustular skin lesions erupted on the chest and abdomen that ulcerated within 2 days. Painful ulcerated lesions with bluish undermined edges were also noted at the left hip wound and two pressure areas of the buttocks. A clinical diagnosis of PG was made and treatment was started with high-dose corticosteroids, which did not lead to improvement. The patient’s past medical history included left eye central retinal vein occlusion with recurrent uveitis and bilateral sensorineural deafness. A diagnosis of atypical CS was made. Four pulsed cyclophosphamide infusions and hyperbaric oxygen healed the lesions. This case demonstrates the complex interplay between PG and CS, which requires further research as it can result in significant morbidity.

## Teaser

Pyoderma gangrenosum can occur in Cogan’s syndrome. This case highlights its association in skin of colour.

**What is already known about this topic?**

- Pyoderma gangrenosum is a very rare association with Cogan syndrome.

**What does this study add?**

- The patient presented with an aggressive form of pyoderma gangrenosum associated with thrombotic manifestations, not previously reported, and this is the first report in a patient with skin of colour.
- This case serves to increase awareness of this rare association with pyoderma gangrenosum.

## Case report

A 75-year-old South Asian woman presented acutely to the Stroke Unit with a left-sided facial droop and limb weakness. Brain magnetic resonance imaging (MRI) revealed a right lacunar infarction, which was managed medically with aspirin and clopidogrel. An unhealed painful infected wound with skin necrosis was noted at the site of a recent total left hip replacement for avascular necrosis of unknown cause. The enlarging nonhealing wound prompted treatment with intravenous antibiotics and multiple surgical debridement under Orthopaedics. An isolated raised alkaline phosphatase was investigated with computed tomography and ultrasound of the abdomen and pelvis. These revealed splenomegaly and a lateral splenic vein thrombosis which were treated with rivaroxaban.

**Figure 1:**
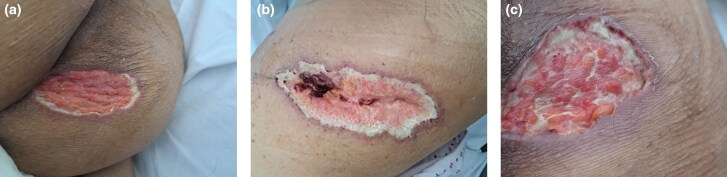
Photos of pyoderma gangrenosum on initial presentation. (a) Lesion on the lower right buttock; (b) lesion on the left hip; (c) lesion on the left upper buttock.

During admission, two new painful pustular skin lesions erupted on the patient’s chest and abdomen. The left hip wound, as well as lesions found on the pressure areas of the buttocks, all appeared ulcerative in nature with bluish undermined edges and were extremely painful (Figure 1a–c). The pustular lesions developed into necrotic ulcers within 2 days. Tissue histology from debridement showed ­nonspecific chronic inflamed tissue and fat necrosis. An incisional biopsy was not done due to a rapidly enlarging hip replacement wound and other painful enlarging ulcers.

Dermatological advice was sought and, based on the lesions’ classical clinical features, a diagnosis of pyoderma gangrenosum (PG) was made. Other diagnoses were excluded with extensive negative autoantibody tests, vasculitis screen, skin swabs, serological tests for syphilis and other atypical infections such as *Coxiella burnetti*, *Bartonella* and *Brucella*. Blood cultures were also negative. High-dose prednisolone (40 mg daily) was commenced which halted the development of new pustules and healed the papulonecrotic skin lesions, but the large ulcers showed very little improvement.

The patient’s past medical history included left eye central retinal vein occlusion (CRVO) with recurrent uveitis of unknown cause causing intermittent visual blurring which was treated with an intravitreal (IVI) steroid implant, IVI ­antivascular endothelial growth factor injections and steroid eye drops. Furthermore, the patient also had a history of sudden vertigo, tinnitus and hearing loss which progressively worsened despite treatment with intratympanic steroid injections. A diagnosis of bilateral sensorineural deafness was established, and a cochlear implant was offered. MRI of the internal auditory meatus and both orbits did not show an organic cause of disease. A provisional diagnosis of atypical Cogan syndrome (CS) with co-existing PG was made due to the presence of audiovestibular dysfunction with inflammatory ocular disease.

CS is a rare autoimmune vasculitis affecting the audio­vestibular and ocular systems.^1^ It is commonly reported in White adults with a mean age of onset of 29 years old.^2^ The pathogenesis is unknown, but it has been proposed to arise from antibody cross-reaction due to viruses.^2^ Haynes *et al*. classified CS in 1980 as typical and atypical.^3^ Typical CS is characterized as ophthalmic disease caused mainly by nonsyphilitic interstitial keratitis (IK) with features of audiovestibular dysfunction within 2 years. This audiovestibular dysfunction must present as sudden-onset Ménière-like attacks, including vertigo, tinnitus and vomiting, and often progressive hearing loss. Atypical CS, in contrast, includes a significant inflammatory ophthalmic disease with or without IK with audiovestibular symptoms that are not characteristic of Ménière-like features and can occur more than 2 years before or after the onset of eye symptoms.^3^

CS is a diagnosis of exclusion, with other key differentials including granulomatosis with polyangiitis, congenital syphilis, Susac syndrome and Takayasu arteritis.^2^ Therefore, extensive investigations were performed for autoantibodies, including antineutrophil cytoplasmic antibody, antinuclear antibody, anti-dsDNA antibody, anti-cyclic citrullinated peptide, complement levels, human leucocyte antigen B27, anti-phospholipid antibody screen, rheumatoid factor and *Treponema pallidum* antibody levels, all of which were negative. Positron-emission tomography showed no systemic vasculitis. A firm diagnosis of atypical CS was therefore made as the patient had left CRVO with uveitis and audiovestibular symptoms.

Cutaneous signs in CS are rare but are known to occur. Besides PG, other reported skin manifestations have included nonspecific skin rashes, urticarial vasculitis, palpable purpura, nodules and ulceration.^2^

PG occurring with CS has only been documented three times in the literature. In addition, other organ manifestations of CS were also not observed in these reports.^1,4,5^ In our case, the patient had a stroke and splenomegaly, which are known manifestations of CS.^2^ Furthermore, all previous cases of PG and CS in the literature were not in people with skin of colour.^1,4,5^

Treatment of CS can be a challenge as there are no treatment guidelines available due to its rarity.^1^ The general mainstay of treatment is with high-dose corticosteroids, which is based on the treatment of other vasculitides.^1^ It is notable that all previous patients of CS with coexistent PG required other immunosuppressive agents in addition to high-dose corticosteroids. The three previously reported patients were treated successfully individually with oral prednisolone (1 mg kg−1), pulsed intravenous cyclophosphamide infusions and minocycline.^1,4,5^

**Figure 2:**
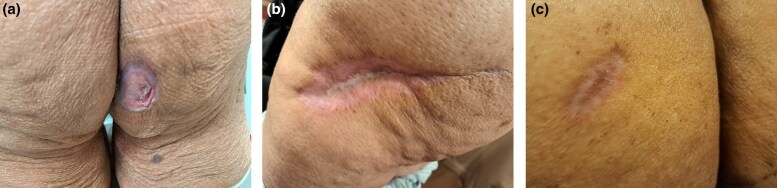
Photos of pyoderma gangrenosum healing from pulsed cyclophosphamide infusions and hyperbaric oxygen therapy. (a) Lesion on the lower right buttock; (b) lesion on the left hip; (c) lesion on the left upper buttock.

As CS is associated with vasculitis, we chose to treat the patient with four 500-mg pulsed cyclophosphamide infusions given at 3-weekly intervals as this has been shown to be effective in treating antineutrophil cytoplasm antibodies-​associated vasculitis.^6^ Four pulsed cyclophosphamide infusions were given in conjunction with hyperbaric oxygen therapy as oral prednisolone failed to halt progression of the disease. This regime successfully controlled the PG ulcerations allowing size reduction, epithelialization and complete healing of all the lesions within 6 months (Figure 2a–c). Low-dose corticosteroids are being continued, with a view to stopping them over the next 3 months.

This case displays the complexity in the diagnosis and treatment of a multisystem disease with PG occurring with CS. Early referral to Dermatology is recommended as our patient demonstrates how PG and CS can result in significant morbidity. The diagnosis of PG was made clinically and after excluding other diagnoses by one of the authors (S.W.) who has expertise in this condition.^7^

## Data Availability

The data underlying this article will be shared on reasonable request to the corresponding author.
